# A Pilot Study Beyond Ovarian Reserve: How AMH Levels Shape the Proteomic and Metabolic Landscape of the Follicle

**DOI:** 10.1002/rmb2.70099

**Published:** 2026-09-22

**Authors:** Rosamaria Militello, Bianca Bartoloni, Gabriella Pinto, Serena Camerini, Angela Amoresano, Francesca Paola Luongo, Paola Piomboni, Alice Luddi, Sara Marchiani, Tania Fiaschi, Tania Gamberi

**Affiliations:** ^1^ Department of Experimental and Clinical Biomedical Sciences “Mario Serio” University of Florence Florence Italy; ^2^ Department of Chemical Sciences University of Naples Federico II Naples Italy; ^3^ Core Facilities Istituto Superiore Di Sanità Rome Italy; ^4^ Department of Molecular and Developmental Medicine University of Siena Siena Italy; ^5^ Departmental Faculty of Medicine Saint Camillus International University of Health Sciences Rome Italy

**Keywords:** adiponectin, anti‐Müllerian hormone (AMH), follicular microenvironment, lactate, mass spectrometry‐based proteomics, ovarian reserve, oxidative stress

## Abstract

**Purpose:**

The follicular fluid (FF) microenvironment critically influences oocyte development, with Anti‐Müllerian Hormone (AMH) serving as a primary marker of ovarian reserve. This exploratory pilot study investigates the biochemical and proteomic shifts across the AMH spectrum to provide initial insights into potential targeted intrafollicular strategies.

**Methods:**

FF samples from 49 patients undergoing assisted reproduction were grouped into Low‐, Normal‐, and High‐AMH groups. Oxidative stress, antioxidant capacity, and metabolic indicators (lactate, adiponectin) were analyzed. Proteomic profiling was performed via LC‐ESI‐MS/MS, followed by STRING network and Spearman correlation analyses.

**Results:**

Distinct molecular shifts occurred across groups. The High‐AMH group showed a marked oxidative imbalance. Conversely, the Low‐AMH group exhibited a distinct signature with higher lactate, lower adiponectin, and downregulated immunoglobulins, alongside upregulated HRG, ITIH3, and GPLD1. Positive correlations between adiponectin and immunoglobulins in Low‐AMH subjects suggest that metabolic and immune‐related factors may be coordinately regulated.

**Conclusions:**

This study suggests that AMH levels are associated with specific follicular microenvironment shifts. Low AMH levels appear linked to a distinct profile characterized by immune and metabolic alterations. These findings provide an exploratory foundation for future larger‐cohort investigations into follicular homeostasis.

## Graphical abstract

Biochemical and proteomic shifts across Anti‐Müllerian Hormone (AMH) categories in human follicular fluid. Follicular fluid from 49 patients undergoing assisted reproduction was stratified by AMH levels (< 0.9, 0.9–2.9, > 3.0 ng/mL). High‐AMH samples displayed marked oxidative imbalance (elevated d‐ROMs). Conversely, the Low‐AMH phenotype exhibited elevated lactate, reduced adiponectin, downregulated immunoglobulins, and upregulated HRG, ITIH3, and GPLD1, highlighting distinct microenvironmental shifts across ovarian reserve phenotypes.

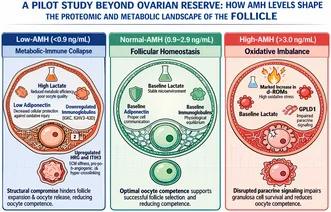

**Abbreviations**
AMH: Anti‐Müllerian hormone
FF: follicular fluid
HAMH: High‐AMH
LAMH: Low‐AMH
NAMH: Normal‐AMH
PCOS: polycystic ovary syndrome

## 1 Introduction

The follicular microenvironment influences the regulation of oocyte development and maturation; therefore, changes in the follicular microenvironment regulate oocyte selection [1]. Granulosa cells support the oocyte's metabolic activity and send signals that regulate its development. Furthermore, the oocyte sends signals to the granulosa cells to regulate their differentiation. This two‐way communication dynamically reshapes the follicular microenvironment, thus ensuring that ovulation releases a fully formed oocyte capable of developing into a healthy embryo [2].

Understanding the complex interactions between the follicular environment and developing oocytes will provide deeper insight into the causes of female infertility [3].

Anti‐Müllerian hormone (AMH) is a protein produced by granulosa cells within small, growing ovarian follicles. It is considered a “thermometer” of female fertility because it provides a direct estimate of ovarian reserve, or the number of eggs still available in the ovaries [4]. One of its advantages is that its levels do not fluctuate significantly throughout the month, allowing the sample to be taken on any day of the menstrual cycle. It also reflects the number of antral and pre‐antral follicles, offering a more precise view of the oocyte population than basal hormone levels alone. AMH measures the quantity, not the quality, of oocytes and is a key parameter in assisted reproduction (ART) programs for predicting ovarian response to medications and personalizing stimulation doses [4]. The literature reports values that indicate an adequate ovarian reserve for a woman's fertile age (1.0–3.5 ng/mL). Lower values (< 1.0 ng/mL) indicate a reduced reserve, while high values (> 5.0 ng/mL) are often associated with polycystic ovary syndrome (PCOS), indicating a very high number of small follicles [5].

Another important molecule in female fertility is adiponectin, a protein hormone mainly secreted by adipose tissue. It regulates ovarian steroidogenesis, oocyte maturation, and embryonic development in humans [6]. It has been measured in the follicular fluid (FF), and several authors have detected its expression in cumulus and granulosa cells. Furthermore, in the human ovary, and more specifically in granulosa cells, adiponectin increases steroid production, which accumulates in the FF; the hormonal content is likely associated with the quality of oocyte maturation, fertilization, implantation rate, and embryonic development [7]. Steroid profiles in stimulated FF are often associated with in vitro fertilization (IVF) prognosis. Oxidative stress also plays a role in the reduction of women's reproductive potential as they age. Increased oxidative stress may mediate the reduced follicle‐stimulating hormone response and poor oocyte quality associated with aging.

In previous studies conducted in our laboratory analyzing FF from women undergoing in vitro fertilization, we determined the metabolic profile, lipid profile, adiponectin, and oxidative stress, and correlated all the obtained parameters with the age of the women analyzed [8]. As a result, we found increased oxidative stress due to both increased androgens and lipid accumulation in the follicular environment, and we concluded that this could be one of the causes of reduced fertility in women over 35 years of age [9].

Within the FF, lactic acid plays a complex dual role in female fertility, functioning both as an essential energy source for the maturing egg and as a metabolic indicator of follicular health and potential dysfunction [10]. While essential for normal development, imbalanced levels of lactic acid are associated with specific infertility conditions such as PCOS and premature aging. Studies indicate that androgen excess (hyperandrogenism) in PCOS causes an accumulation of lactic acid, leading to acidification within the follicles [11]. However, cumulus cells surrounding the oocyte convert glucose into pyruvate and lactate. The oocyte takes up this lactate and converts it into energy essential for maturation [12]. Moreover, high levels of lactate are generally associated with healthy follicle growth, while reduced levels may indicate developmental arrest. On the contrary, a significant increase in lactate within FF can be part of a broader, high‐stress follicular environment that causes chronic inflammation, negatively affecting fertility [13].

The aims of this exploratory, discovery‐phase pilot study are: (*a*) to quantify adiponectin, lactate, and oxidative stress levels as a function of anti‐Müllerian hormone (AMH) concentrations in FF in women undergoing in vitro fertilization (IVF), (*b*) to identify their proteomic profile, and (*c*) to correlate the information obtained. To achieve this, the cohort was stratified into three distinct clinical groups based on established AMH cut‐off points representing diminished, normal, and high ovarian reserves (Low‐AMH < 0.9 ng/mL; Normal‐AMH 0.9–2.9 ng/mL; High‐AMH > 3.0 ng/mL) [14]. We hypothesized that these distinct AMH categories could reflect specific biochemical shifts in the follicular microenvironment and proteomic signature, which might be associated with variations in oocyte development and maturation. Identifying these parameters may provide a deeper understanding of the molecular factors influencing follicular physiology and microenvironmental dynamics. It will also be important to evaluate proteins linked to hormonal variation and whether these could serve as potential targets for future investigative research. Ultimately, elucidating these group‐specific molecular signatures could provide a foundation for future, larger‐cohort studies investigating potential non‐invasive biomarkers to explore the follicular microenvironment, providing preliminary hypotheses on localized homeostatic mechanisms.

## 2 Material and Methods

The d‐ROM Test and the BAP Test kit were obtained from Diacron International s.r.l. (Grosseto, Italy), the Adiponectin ELISA Assay kit from Adipogen Life Sciences (Liestal, Switzerland) and the K‐LATE kit from Megazyme (Bray, Ireland).

### 2.1 Follicular Fluids Collection and Analysis

Follicular fluids were collected as discarded material during oocyte retrieval procedures following cumulus‐oocyte complex (COC) isolations in patients undergoing medically assisted reproduction (MAR) at the MAR Unit of Siena University Hospital (Italy). A total of 49 samples were collected from women aged 30–42 with no reported health issues, all of whom were non‐smokers and non‐drinkers. All participants volunteered for the study after providing informed consent. The study was compliant with the Declaration of Helsinki and approved by the ethics committee of the University of Siena (CEAVSE ethics statement, Prot. No. 11161_2017).

On the day of oocyte retrieval, FF samples were centrifuged at 2 500 *g* for 10 min at 4°C and subsequently stored at −80°C and processed as reported in Luti et al. [8]. The 49 samples were divided into three groups based on the anti‐Müllerian hormone (AMH) levels: 16 called Low‐AMH (LAMH) who had a hormone value < 0.9 ng/mL; 20 called Normal‐AMH (NAMH) with a value from 0.9 to 2.9 ng/mL; and 13 classified as High‐AMH (HAMH) with a value > 3 ng/mL. This grouping and the baseline demographic and clinical characteristics of the cohort (*n* = 49) are summarized in Table 1. Baseline demographic characteristics and the distribution of infertility etiologies were compared across the three groups. Given the exploratory nature of this discovery‐phase pilot study, our sample size of 49 subjects can be considered appropriate for the identification of biochemical and proteomic shifts, aligning with current literature standards for high‐resolution mass spectrometry and proteomic assays.

**TABLE 1:** Demographic and clinical characteristics stratified by AMH group.

Characteristics	LAMH (*n* = 16)	NAMH (*n* = 20)	HAMH (*n* = 13)	*p*
Age (years)^a^	39.3 ± 3.6	35.4 ± 7	33.8 ± 5.5	* LAMH vs HAMH
AMH (ng/mL)	0.5 ± 0.3	2.6 ± 0.2	5.3 ± 1.7	****All
Male infertility factor, *n* (%)	12.5% (2/16)	30% (6/20)	30.8% (4/13)	—
Female infertility factor, *n* (%)	68.8% (11/16)	65% (13/20)	61.5% (8/13)	—
Diminished ovarian reserve (%)	68.8% (11/16)	5% (1/20)	0% (0/13)	—
Endocrine–ovulatory disorders (%)	12.5% (2/16)	30% (6/20)	53.8% (7/13)	—
Tubal factor (%)	0% (0/16)	5% (1/20)	15.4% (2/13)	—
Multiple female factors (%)	0% (0/16)	10% (2/20)	0% (0/13)	—
Idiopathic infertility (%)	0% (0/16)	10% (2/20)	0% (0/13)	—
Pre‐treatment fertility preservation/social oocyte freezing (%)	0% (0/16)	10% (2/20)	0% (0/13)	—
Unspecified/no female factor reported (%)	18.8% (3/16)	10% (2/20)	15.4% (2/13)	—

*Note:* Data are presented as mean ± standard deviation (SD) or percentage, as appropriate. GraphPad Prism 8 was used for data analysis, and Tukey's test was performed (*p < 0.05; ****p < 0.0001).

Abbreviations: AMH, anti‐Müllerian hormone; HAMH, high anti‐Müllerian hormone; LAMH, low anti‐Müllerian hormone; NAMH, normal anti‐Müllerian hormone.

^a^ Age variation across AMH groups is expected, as AMH physiologically declines with advancing female age [4].

### 2.2 Oxidative Stress Measurements

The d‐ROM test (diacron Reactive Oxygen Metabolites) and the BAP test (Biological Antioxidant Potential) were used to assess the levels of reactive oxygen metabolites and the antioxidant capacity in FF. The parameters d‐ROMs and BAP were selected based on their long‐term stability, and the measurements were carried out according to the manufacturer's instructions and as previously reported in Militello et al. [15].

The d‐ROM test provides an indirect measure of oxidative stress by quantifying organic hydroperoxides, which are generated from reactive oxygen species. Briefly, biological samples are dissolved in an acidic buffer, which promotes the release of metal ions (Fe) from sample proteins. In the presence of iron, hydroperoxides are converted into alkoxyl and peroxyl radicals. These newly formed radicals subsequently oxidize an additive substituted aromatic amine (N, N‐diethyl‐para‐phenylenediamine), leading to the formation of a relatively stable, pink‐colored cation radical. The resulting chromogen is quantified spectrophotometrically at 505 nm, and the absorbance is directly proportional to the concentration of reactive oxygen metabolites in the sample.

The BAP test provides an estimate of the total antioxidant capacity of a biological sample by assessing its overall reducing potential against ferric (Fe^3+^) ions. The assay is based on a colored solution containing ferric ions complexed with a chromogenic thiocyanate derivative. Analyses were carried out using a free radical analyzer system (FREE Carpe Diem, DIACRON INTERNATIONAL s.r.l.), equipped with a spectrophotometric device reader and a thermostatically controlled microcentrifuge. The d‐ROM results are expressed in arbitrary units (UCARR), where 1 UCARR corresponds to 0.8 mg/L of hydrogen peroxide. The BAP results are expressed as μmol/L of reduced ferric ions.

### 2.3 Adiponectin Measurement

Total adiponectin concentrations were determined using a commercially available human Adiponectin Enzyme‐Linked Immunosorbent Assay (ELISA) kit (Adipogen Life Sciences, Liestal, Switzerland). Briefly, this assay employs specific monoclonal antibodies to quantitatively detect adiponectin in biological samples. All reagents, standards, and controls were used according to the manufacturer's instructions, and optical density was measured at the recommended wavelength to calculate concentrations based on the standard curve. The assay has a sensitivity of 100 pg/mL, defined as the lowest detectable concentration, with a dynamic detection range from 0.5 to 32 ng/mL.

### 2.4 Lactate Assay

The measurement of lactate levels in FF samples was performed using the K‐LATE kit (Megazyme, Bray, Ireland) according to the manufacturer's instructions and as previously reported in Mannelli et al. [16]. The assay quantifies the increase in NADH concentration generated during the reaction, which is linear within a range of 0.3–3.0 μg of L‐lactic acid per assay. Absorbance was measured at 340 nm immediately before and 10 min after the addition of L‐LDH. Lactate concentrations were normalized to the total protein content of each sample.

### 2.5 Statistical Analysis of Biochemical Assays

All biochemical measurements were executed by laboratory personnel blind to the clinical characteristics and AMH group assignment of the patients. Follicular fluid samples were randomized and identified only by alphanumeric codes to prevent investigator bias. For the adiponectin assay, the intra‐ and inter‐assay coefficients of variation (CV) were < 5.2% and < 7.5%, respectively. For the lactate determination, intra‐ and inter‐assay CVs were < 4.0% and < 6.8%. For the oxidative stress markers, the intra‐assay CV was < 6.0% and the inter‐assay CV was < 8.5%. All biochemical assays were performed in duplicate for each FF sample to ensure reproducibility and minimize experimental error. Data are expressed as means ± standard deviation (SD). Statistical analysis was performed using one‐way analysis of variance (ANOVA) followed by Tukey's multiple comparisons test using GraphPad Prism version 8.0.2 (GraphPad Software, San Diego, CA, USA). Tukey's test was used to compare all possible pairs of group means, applying a family‐wise confidence level of 95%. A *p*‐value < 0.05 was considered statistically significant (* *p* < 0.05; ** *p* < 0.01; *** *p* < 0.001; **** *p* < 0.0001).

Correlations were performed using Spearman's rank correlation in GraphPad Prism, and *p*‐values were adjusted for multiple testing using the Benjamini–Hochberg false discovery rate (FDR) correction, with an adjusted *p*‐value (q‐value) < 0.05 considered statistically significant.

### 2.6 Sample Preparation for Liquid Chromatography‐Electrospray Ionization‐Tandem Mass Spectrometry (LC‐ESI‐MS/MS) Analysis

Following protein quantification by the Bradford assay, 500 μg of FF proteins were isolated via chloroform/methanol precipitation (4:1:3 v/v/v methanol/chloroform/water). The air‐dried pellet was solubilized in 25 mM ammonium bicarbonate (AMBIC) with 6 M urea, reduced using 20 mM DTT (1 h, 60°C), and alkylated with 40 mM iodoacetamide (1 h, room temperature, dark). The solution was diluted with 25 mM AMBIC to < 1 M urea and digested overnight at 37°C with trypsin (1:50 enzyme‐to‐substrate ratio). Proteolysis was quenched after 16 h using 10 μL of 10% formic acid.

### 2.7 LC‐ESI‐MS/MS Analysis

Each peptide mixture was analyzed in triplicate using an Ultimate 3000 ultra‐high‐performance liquid chromatography (UHPLC) system (Thermo Fisher Scientific, San Jose, CA, USA) connected to an Orbitrap Fusion Tribrid mass spectrometer (Thermo Fisher Scientific, CA, USA). Desalting was performed online using a trap column (Acclaim PepMap 100 C18, Thermo Fisher Scientific), followed by chromatographic separation on a 45 cm capillary column (ICT 36 007 508–50‐5, MS WIL, Aarle‐Rixtel, The Netherlands). The column was kept at 40°C using a Sonation column oven and interfaced with a Nanospray Flex ion source (Thermo). Peptides were eluted over a 95‐min gradient using two solvents: buffer A (95% H_2_O, 5% acetonitrile, 0.1% formic acid) and buffer B (95% acetonitrile, 5% H_2_O, 0.1% formic acid), delivered at a flow rate of 250 nL/min. The mass spectrometer was operated in positive‐ion mode using data‐dependent acquisition (DDA). Full MS scans (MS1) were acquired in the Orbitrap at a resolution of 120 000, covering a mass‐to‐charge (m/z) range of 350–1 550. Within each acquisition cycle (3 s max), the ten most intense precursor ions with charge states above +1 were isolated using the monoisotopic precursor selection (MIPS) filter and subjected to higher‐energy collisional dissociation (HCD) at 30% normalized collision energy. The resulting fragment ions were analyzed in the ion trap using a rapid scan setting.

### 2.8 Database Search and Protein Identification

Raw data files from the MS runs were processed using the Andromeda search engine within the MaxQuant bioinformatics suite [17], searching against the *Homo sapiens* FASTA file download from the UniProt database. Identification parameters included a minimum peptide length of 6 amino acids and a minimum of 1 peptide (both razor and unique peptides). The mass tolerance was set to 10 ppm for precursor ions and 0.6 Da for fragment ions. Trypsin was specified as the proteolytic enzyme, with cysteine carbamidomethylation set as a fixed modification. Variable modifications included methionine oxidation and N‐terminal pyroglutamate formation from glutamine. Peptide‐spectrum matches (PSMs) were filtered using the target‐decoy approach, applying a peptide‐level false discovery rate (FDR) of 1% (e‐value ≤ 0.01), corresponding to a 99% confidence level.

### 2.9 Statistical Analysis of the Proteomics Data

Statistical analyses of the proteomics data were performed using Perseus [18]. Data were initially evaluated for normality and homogeneity of variance using standard diagnostic tests. In differential proteomics experiments, normalization begins at the sample preparation stage. Before enzymatic digestion, equal amounts of total protein were processed for each sample. This step is particularly important because the experimental treatments may induce changes in the overall protein content of the samples. By starting with equal amounts of protein, the observed differences in protein abundance are more likely to reflect dysregulation associated with the physiological condition rather than differences in sample loading. A second normalization step was performed during data processing in MaxQuant using the MaxLFQ (label‐free quantification) algorithm. MaxLFQ calculates protein abundances by first determining peptide intensity ratios between all pairs of samples in which a peptide is quantified. These pairwise peptide ratios are then combined through a least‐squares optimization approach to generate normalized protein intensities across the entire dataset. This procedure minimizes systematic technical variation, such as differences in sample loading and LC–MS signal intensity, while preserving real biological differences in protein abundance. MaxLFQ generates protein intensity values that are directly comparable across samples without the need for external standards. Moreover, protein filtering was performed using the following criteria: (i) reverse protein entries were removed to avoid the inclusion of false‐positive identifications generated during database searching; (ii) known contaminant proteins were excluded from the analysis; and (iii) proteins were retained only if they contained at least three valid quantitative values across the dataset. LFQ intensities obtained from MaxQuant were imported into Perseus and log2‐transformed. Missing values were imputed from a normal distribution (width = 0.3, down shift = 1.8) to represent proteins with abundances below the detection limit. Subsequent statistical analyses were performed on the filtered and imputed dataset. Differences among experimental groups were assessed by one‐way analysis of variance (one‐way ANOVA). When a significant overall effect was observed, post hoc multiple comparisons were carried out using Tukey's honestly significant difference (HSD) test to identify pairwise differences between groups. The output of this analysis included nominal *p*‐values alongside log_2_ fold‐change values, calculated as the ratio of the mean values between each pair of conditions. To control for multiple testing, these pairwise comparisons were subsequently corrected using the Benjamini–Hochberg False Discovery Rate (FDR) method, defining statistical significance at an adjusted *p*‐value (q‐value) < 0.05. Only proteomic findings that remained significant after FDR correction are reported as statistically significant in Table 2.

**TABLE 2:** Differentially abundant proteins identified by LC‐ESI‐MS/MS analysis among the three AMH groups: LAMH, NAMH, and HAMH.

Protein name	Accession number^a^	Gene name	Peptides^b^	Sequence coverage [%]^c^	Score	Tukey's test^d^/ln2 fold change^e^		
						HAMH vs LAMH	HAMH vs NAMH	LAMH vs NAMH
Immunoglobulin heavy variable 3‐43D	P0DP04	IGHV3‐43D	3	34.7	15.04	***/1.3	ns	***/−1.7
Keratin, Type I cuticular Ha6	O76013	KRT36	1	3.2	3.16	**/−1.8	ns	**/2.9
Immunoglobulin kappa constant	P01834	IGKC	26	100	323.31	**/0.7	ns	**/−0.8
ATPase family AAA domain‐containing protein 2	Q6PL18	ATAD2	1	1.2	3.26	ns	ns	**/−1.2
Histidine‐rich glycoprotein	P04196	HRG	18	33	313.92	**/−0.8	ns	ns
Immunoglobulin kappa variable 3–15	P01624	IGKV3‐15	2	26.1	57.84	ns	ns	**/−1.4
Hemoglobin subunit gamma‐2	P69892	HBG2	6	49.7	13.01	ns	**−2.1	ns
Immunoglobulin heavy variable 4–59	P01825	IGHV4‐59	2	26.7	323.31	ns	ns	**−1.9
Actin, cytoplasmic 2	P63261	ACTG1	11	34.4	171.13	*‐2.6	ns	ns
Inter‐alpha‐trypsin inhibitor heavy chain H3	Q06033	ITIH3	10	18.1	109.32	*‐1	ns	ns
Immunoglobulin heavy constant mu	P01871	IGHM	20	47.7	323.31	*‐1.4	ns	ns
Immunoglobulin kappa variable 2–29	A2NJV5	IGKV2‐29	2	16.7	32.89	ns	ns	*−1.2
Metalloproteinase inhibitor 1	P01033	TIMP1	4	34.8	71.98	*1	ns	ns
Phosphatidylinositol‐glycan‐specific phospholipase D	P80108	GPLD1	3	6.8	3.07	*−0.5	ns	ns

^a^ UniProt accession number.

^b^ Number of unique peptides used for protein identification.

^c^ Coverage = (number of identified residues/total number of amino acid residues in the protein sequence) × 100%.

^d^ Tukey's test was performed by Perseus; symbols (*p < 0.05; **p < 0.01; ***p < 0.001) indicate significant pairwise differences for proteins that remained statistically significant after Benjamini−Hochberg False Discovery Rate (FDR) correction (q < 0.05) in the proteomic analysis (ns = not significant).

^e^ Log_2_ fold change values. Fold changes were calculated as the ratio of the mean values between each pair of conditions: LAMH, low anti‐Müllerian hormone; NAMH, normal anti‐Müllerian hormone; HAMH, high anti‐Müllerian hormone. It was reported only for statistically significant values.

While high‐throughput proteomic studies are inherently subject to biological variability, a sample size of *n* = 49 individual patients is robust and fully aligned with several published discovery‐phase proteomic investigations of human follicular fluid, which typically feature cohorts of 20 to 40 subjects due to cost and technical constraints. This cohort can be considered appropriate for the exploratory nature of this discovery‐phase pilot study to identify major differences and exploratory associations between distinct clinical phenotypes (Low vs. Normal vs. High AMH), consistent with current literature standards for high‐resolution mass spectrometry/targeted proteomic assays [19].

### 2.10 Graphical Abstract Preparation

The graphical abstract was generated using Microsoft Copilot (Microsoft Corporation, Redmond, WA, USA). The figure was designed to illustrate the proteomic and metabolic differences among follicular groups classified by Anti‐Müllerian Hormone (AMH) levels (Low < 0.9 ng/mL, Normal 0.9–2.9 ng/mL, High > 3.0 ng/mL). The original schematic layout was preserved, while English terminology and color palette were refined for journal‐ready clarity. No generative data were introduced; all biological content derives from the study's experimental results.

## 3 Results

### 3.1 Oxidative Stress and Antioxidant Capacity Measurements in LAMH, NAMH, and HAMH Follicular Fluids

**FIGURE 1:**
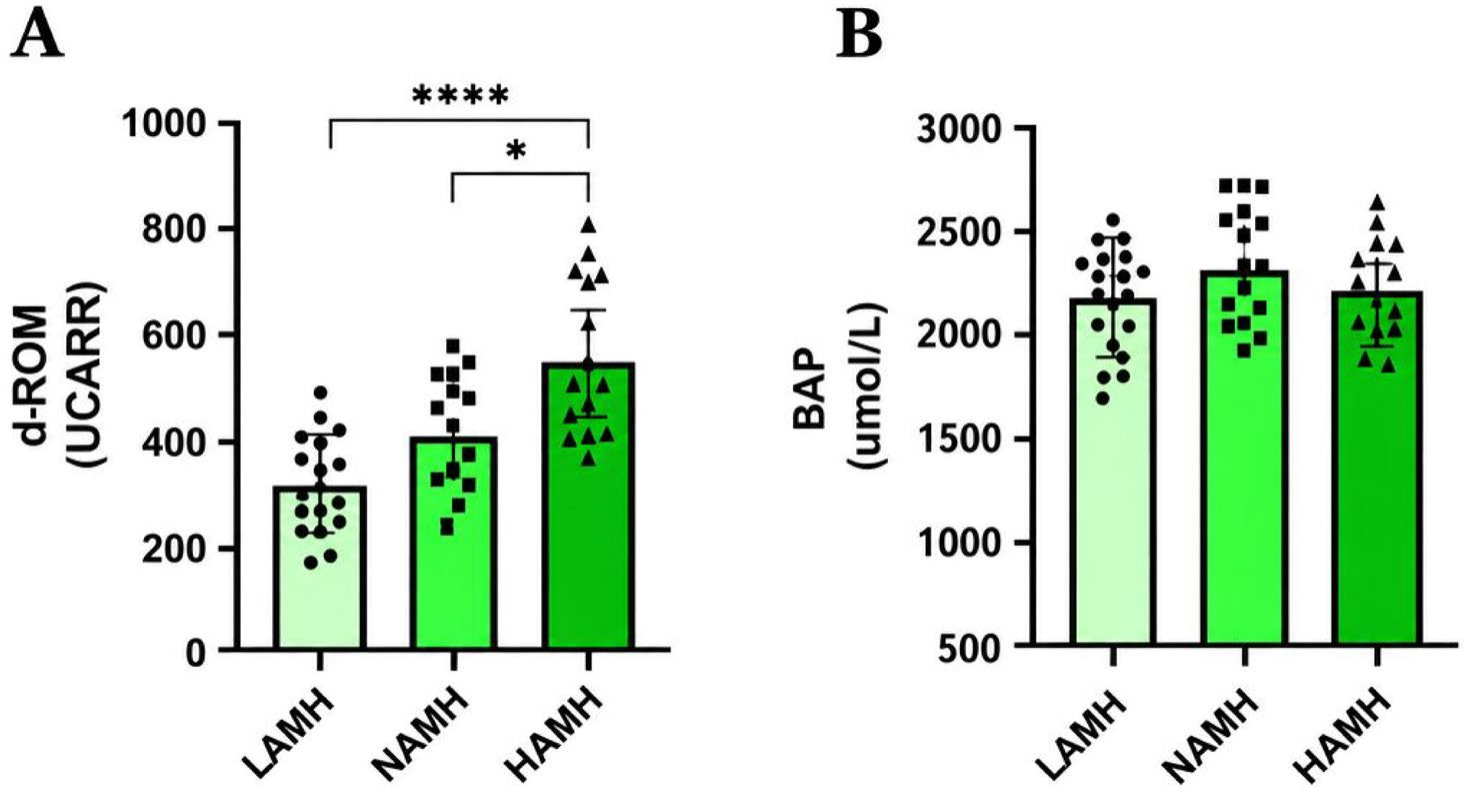
Follicular fluid oxidative stress determination. (A) Reactive oxygen metabolites were measured by the d‐ROM test (derivatives of Reactive Oxygen Metabolites). (B) Antioxidant capacity was assessed by the BAP test (Biological Antioxidant Potential) using a free radical analyzer system (FREE Carpe Diem, DIACRON INTERNATIONAL s.r.l.). Data were analyzed by one‐way ANOVA followed by Tukey's post hoc test using GraphPad Prism version 8.0.2 (*p < 0.05; ****p < 0.0001).

Oxidative species and antioxidant defenses play a crucial role in cellular metabolism and in maintaining follicular environment homeostasis. Levels of oxidative species (d‐ROM assay) and antioxidant capacity (BAP assay) in FF were analyzed across the three groups (LAMH, NAMH, and HAMH), and the results are shown in Figure 1. A detailed analysis of d‐ROM values (Figure 1a) revealed significantly higher levels in the HAMH group (544.8 ± 153.2 UCARR) compared with the LAMH group (329.1 ± 106.3 UCARR) with a 1.66‐fold increase (*p* < 0.0001) and the NAMH group (428.7 ± 107.5 UCARR), with a 1.27‐fold increase (*p* < 0.05). No significant difference was observed between the LAMH and NAMH groups (fold change 0.77; *p* = 0.0545). In contrast, BAP values (Figure 1b) were comparable across groups, with mean concentrations of 2 177 ± 283.6 μmol/L in the LAMH group, 2 300 ± 252.4 μmol/L in the NAMH group, and 2 207 ± 221.3 μmol/L in the HAMH group. Pairwise comparisons showed no significant differences between LAMH and NAMH (*p* = 0.3537), LAMH and HAMH (*p* = 0.9517), or NAMH and HAMH (*p* = 0.6159).

Overall, these findings indicate that although oxidative stress levels are significantly increased in the HAMH group, antioxidant capacity remains comparable across groups, suggesting a potential imbalance between reactive oxidative species production and antioxidant defense.

### 3.2 Assessment of Lactate Levels in LAMH, NAMH and HAMH Follicular Fluids

**FIGURE 2:**
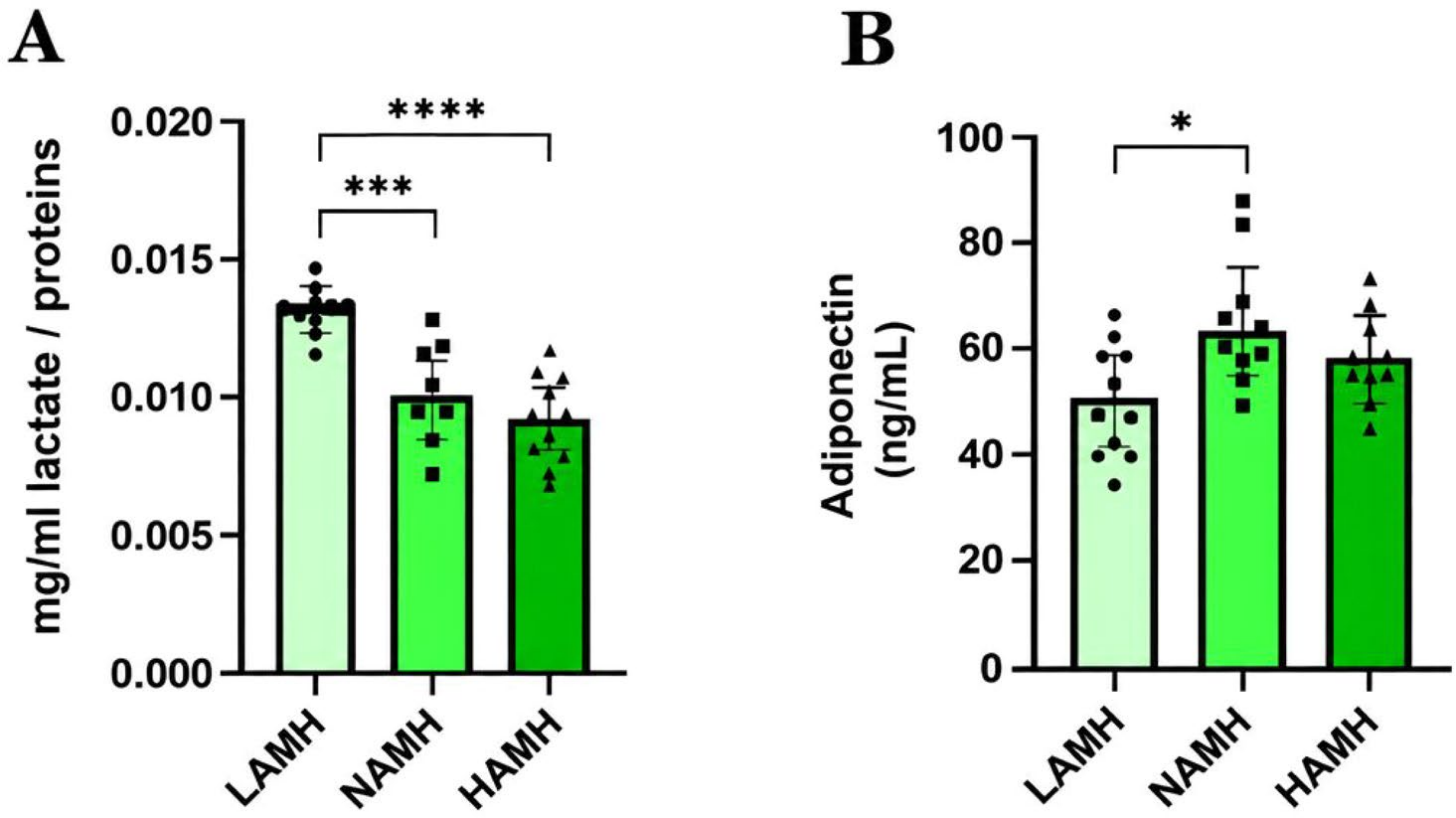
Follicular Fluid determination of: (A) Levels of lactate measured using a lactate assay. Lactate concentrations (mg/mL) were normalized to total protein content. Statistical analysis was performed by one‐way ANOVA using GraphPad Prism version 8.0.2 (***p < 0.001; ****p < 0.0001). (B) Levels of adiponectin were measured using a specific ELISA assay. The concentrations of adiponectin are reported in ng/mL. Statistical analysis was performed by one‐way ANOVA followed by Tukey's post hoc test using GraphPad Prism version 8.0.2 (*p < 0.05).

Lactate is a key metabolite in energy metabolism and may reflect shifts in follicular metabolic activity. Lactate concentrations were evaluated across the three groups, and the results are shown in Figure 2a. The LAMH group exhibited significantly higher lactate levels (0.01320 ± 0.0007828 mg/mL) compared with both the NAMH group (0.0099 ± 0.002191 mg/mL; fold change 1.33; *p* = 0.0002) and the HAMH group (0.0092 ± 0.001381 mg/mL; fold change 1.43; *p* < 0.0001). No significant difference was observed between the normal and HAMH groups (*p* = 0.5443). These findings indicate that elevated lactate levels are specifically associated with the LAMH condition, whereas the normal and HAMH groups exhibit comparable lactate profiles.

### 3.3 Adiponectin Concentrations in LAMH, NAMH and HAMH Follicular Fluids

Adiponectin is an adipokine with anti‐inflammatory and metabolic regulatory functions and has been proposed to play an important role in female reproduction and infertility. FF adiponectin concentrations were evaluated across the LAMH, NAMH, and HAMH groups (Figure 2b). Adiponectin levels were significantly lower in the LAMH group (50.16 ± 10.55 ng/mL) compared with the NAMH group (63.51 ± 12.71 ng/mL; fold change 0.79; *p* = 0.0162). No significant difference was observed between LAMH and HAMH groups (57.71 ± 8.69 ng/mL; *p* = 0.2492) or between normal and HAMH groups (*p* = 0.4478). These findings indicate a selective reduction of adiponectin levels in the LAMH group, whereas the normal and HAMH groups exhibit comparable concentrations.

### 3.4 Proteomic Profile in the LAMH, NAMH and HAMH Groups

**FIGURE 3:**
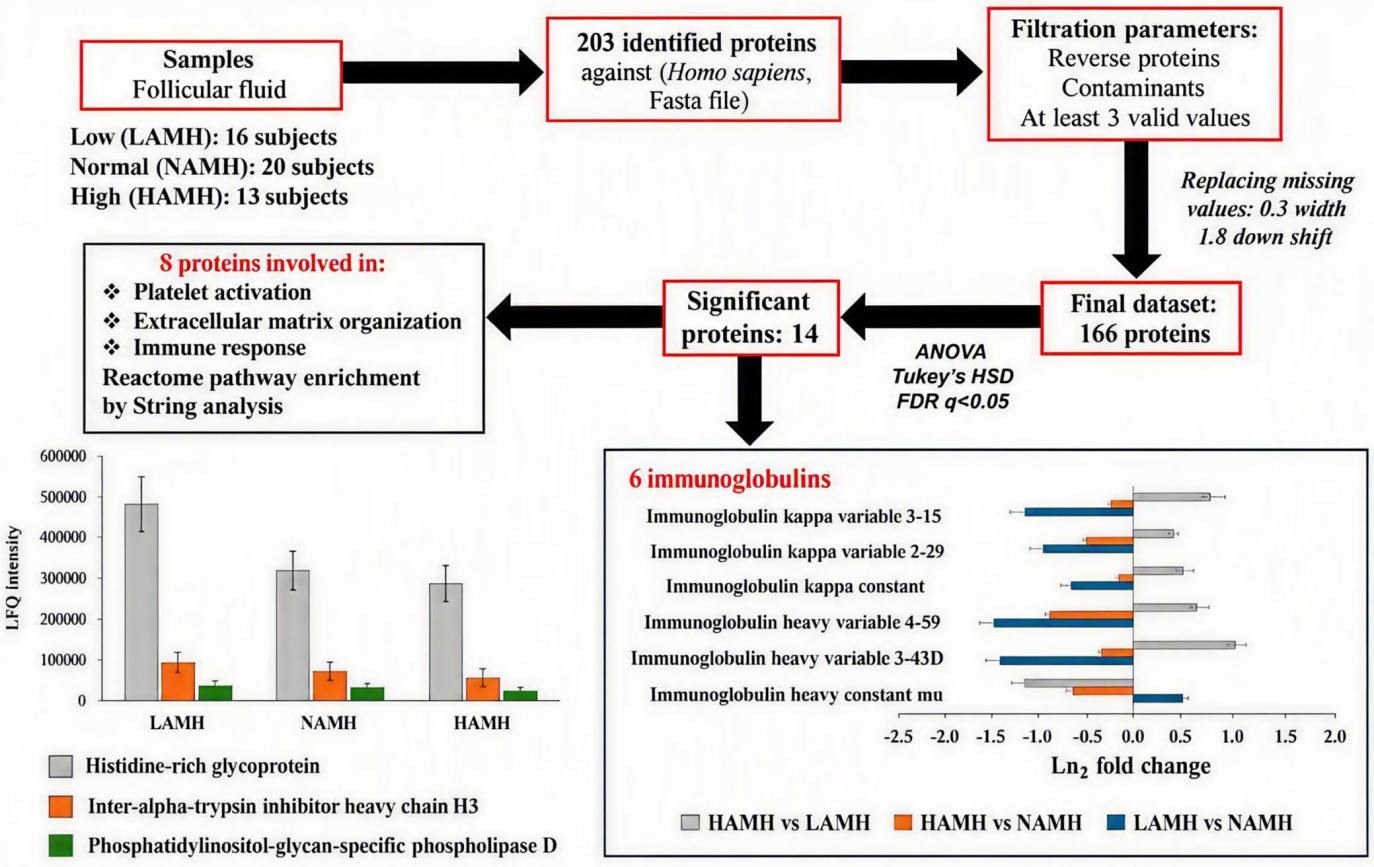
Workflow of the proteomic analysis performed on follicular fluid samples. Samples were grouped according to Low Anti‐Müllerian Hormone (LAMH), Normal Anti‐Müllerian Hormone (NAMH), and High Anti‐Müllerian Hormone (HAMH) levels. A total of 203 proteins were initially identified and subsequently filtered for reverse protein sequences, contaminants, and a minimum of three valid values per group, with missing values imputed using a downshift approach. The final dataset included 166 proteins, of which 14 were found to be significantly different by one‐way ANOVA followed by Tukey's HSD post hoc test and remained statistically significant after Benjamini–Hochberg False Discovery Rate (FDR) correction (*q* < 0.05). Functional enrichment analysis indicated involvement in platelet degranulation and hemostasis, as well as in pathways annotated in Reactome. STRING‐based protein–protein interaction network analysis identified interactions among eight proteins, of which three were particularly relevant, as illustrated in the histogram representation. Among the significant proteins, six immunoglobulins were highlighted, with differential expression patterns observed across AMH groups.

The proteomic profile of FF was obtained using an LC‐ESI‐MS/MS analysis approach. A total of 203 proteins were initially identified from the three FF samples using MaxQuant software (Table S1). After applying quality filtering criteria, 166 proteins (over 80%) were retained for downstream analysis. Among these, 14 proteins were identified as differentially abundant across the three AMH groups (LAMH, NAMH, and HAMH). These proteins were selected based on an initial one‐way ANOVA followed by Tukey's HSD post hoc test, with only those surviving the stringent Benjamini–Hochberg FDR correction (adjusted *p*‐value, *q* < 0.05) being reported in Table 2. Among the 14 proteins showing significant differences, six were immunoglobulins, which were predominantly downregulated in subjects with LAMH compared with the other two groups. This pattern suggests a lower abundance of immunoglobulins in women with diminished ovarian reserve. Among the remaining eight proteins, three formed a functional cluster in STRING network analysis, exhibiting a similar expression pattern characterized by higher abundance in the LAMH group compared with the NAMH and HAMH groups. Specifically, HRG (Histidine‐Rich Glycoprotein), ITIH3 (Inter‐Alpha‐Trypsin Inhibitor Heavy Chain H3), and GPLD1 (Glycosylphosphatidylinositol Specific Phospholipase D1) were significantly upregulated in LAMH samples relative to both NAMH and HAMH (Figure 3).

### 3.5 Correlation Among Anti‐Müllerian Hormone, Adiponectin, Lactate, Oxidative Stress and the Identified Proteins

**FIGURE 4:**
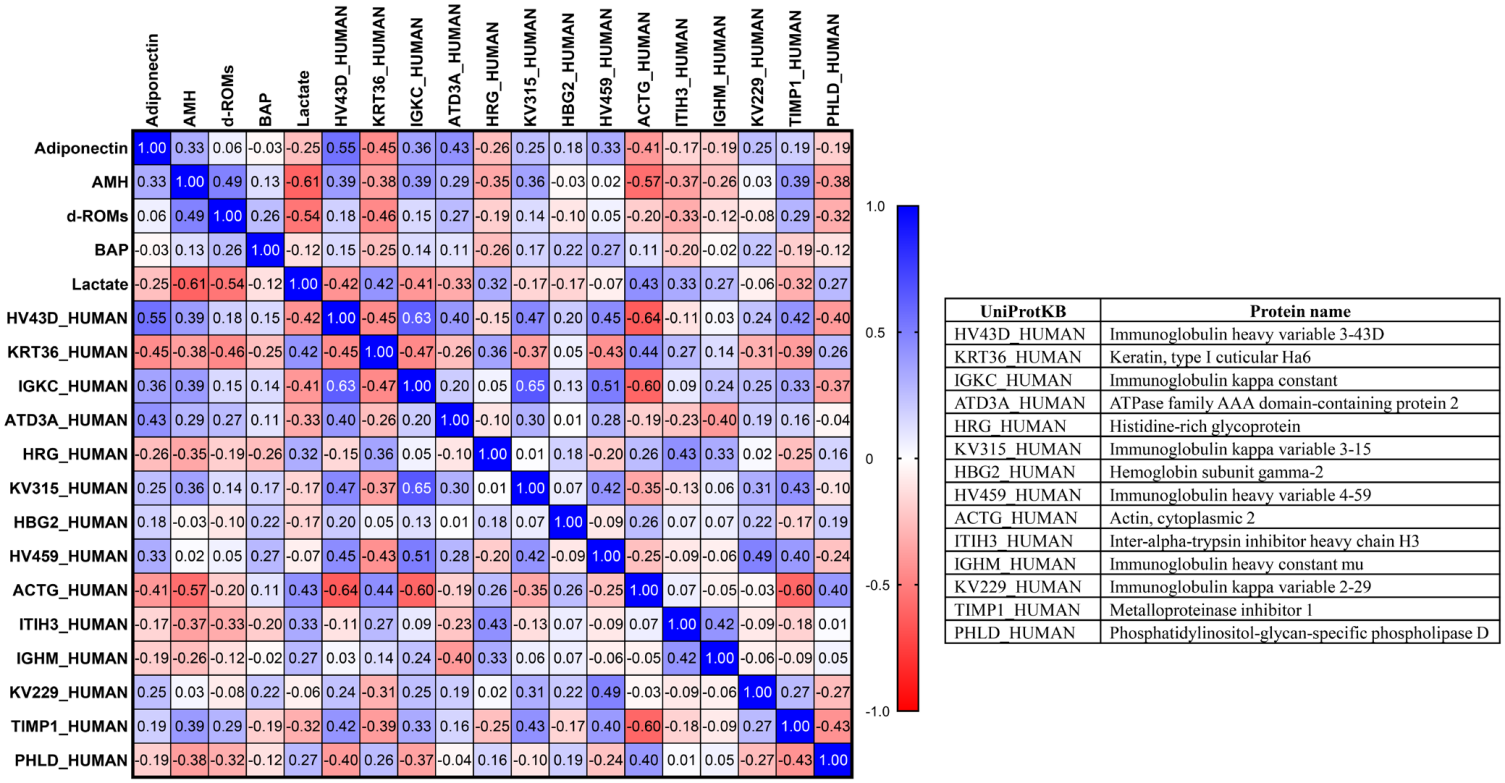
Heatmap of Spearman correlation coefficients among proteins, oxidative stress measurements, and other metabolites. Color intensity indicates the strength of the correlation, with red representing negative correlations and blue representing positive correlations. Significance was defined as an adjusted *p*‐value (*q*‐value) < 0.05 following Benjamini–Hochberg FDR correction.

A heatmap of Spearman correlation coefficients reported in Figure 4 visually displays the strength and direction of linear relationships between metabolites and identified proteins for follicular fluids. In the heatmap, the blue color indicates a positive linear relationship and the red a negative correlation. As reported in Figure 4, adiponectin is negatively correlated with lactate (*r* = −0.25; *p* = 0.148), and shows a positive correlation with two immunoglobulins: Immunoglobulin kappa constant (IGKC; *r* = 0.36; *p* = 0.038), Immunoglobulin heavy variable 3‐43D (IGHV3‐43D; *r* = 0.55; *p* = 0.001). As shown in the figure, adiponectin shows a slight positive correlation with Metalloproteinase inhibitor 1 (*r* = 0.19; *p* = 0.288). AMH is negatively correlated with lactate (*r* = −0.61; *p* < 0.0001) and actin cytoplasmic 2 (*r* = −0.57; *p* < 0.0001); on the contrary, this hormone shows a positive correlation with the reactive oxygen metabolites in FF d‐ROM (*r* = 0.49; *p* = 0.003). d‐ROM shows a negative correlation with lactate (*r* = −0.54; *p* = 0.001) following the same trend than AMH.

## 4 Discussion

The study aimed to analyze the biochemical and proteomic profile of follicular fluid in relation to AMH levels, focusing in particular on adiponectin, lactate, and oxidative stress. The proteomic results revealed a predominant downregulation of immunoglobulins in subjects with LAMH compared to the other two groups. Alterations in immune‐related components of the follicular environment have previously been reported in women with diminished ovarian reserve (DOR) [20]. For example, a study investigating cytokine profiles reported that 59 cytokines exhibited significantly different concentrations in women with DOR compared with controls, with nearly 90% of these cytokines being upregulated [21]. These findings support the concept that the ovarian microenvironment in women with reduced ovarian reserve exhibits alterations in immune‐related pathways. In this context, the observed downregulation of immunoglobulins may reflect modifications in immune regulatory mechanisms involved in maintaining follicular homeostasis. Such changes could contribute to alterations in the local biochemical milieu, potentially affecting the homeostasis of the follicular microenvironment associated with oocyte maturation.

Among the other significantly changing proteins, HRG is a multifunctional plasma protein involved in the regulation of coagulation, angiogenesis, and immune responses. Elevated HRG levels are associated with alterations in follicular health, potentially acting as a potent anti‐angiogenic factor and a modulator of the local inflammatory cascade [22]. Mechanistically, it is hypothesized that excess HRG might interfere with the delicate microvascular remodeling of the theca cell layer required during dominant follicle selection, reducing local oxygen and nutrient delivery to the maturing oocyte [23]. Previous evidence indicates that ovarian response can be influenced by specific HRG polymorphisms, further supporting a functional role for this protein in ovarian physiology.

ITIH3 contributes to extracellular matrix (ECM) stabilization and modulates inflammatory processes. Its upregulation in LAMH samples provides key mechanistic insights into physical and structural alterations within the ovary. ITIH3 is crucial for binding hyaluronan; thus, its excessive upregulation could potentially lead to over‐stabilization and hyper‐crosslinking of the ECM. This increased matrix stiffness might restrict the mechanical expansion of the growing follicle and impair the tissue fluidity necessary for successful ovulation, potentially affecting the physical properties of the follicular niche, which may have implications for oocyte health [19]. Notably, ITIH3 has also been reported as significantly upregulated in follicular fluid in a quantitative label‐free proteomic study identifying candidate biomarkers for endometriosis‐associated infertility.

GPLD1 expression was similar between LAMH and NAMH groups but showed a marked reduction in HAMH subjects, suggesting that downregulation may be specifically associated with high AMH levels rather than diminished or normal ovarian reserve. Given its role in cleaving glycosylphosphatidylinositol (GPI) anchors and regulating the release of GPI‐anchored proteins, the hypothesized consequence of reduced GPLD1 abundance is a potential alteration in shedding of membrane‐bound receptors and signaling molecules [24]. This downregulation could potentially perturb crucial paracrine cell–cell signaling pathways—such as the TGF‐β/BMP superfamily or insulin‐like growth factor pathways, which heavily rely on GPI‐anchored proteins on granulosa cells to dictate follicle survival and prevent premature atresia [25]. The coordinated upregulation of HRG and ITIH3 in LAMH, along withthe selective downregulation of GPLD1 in HAMH, highlights distinct alterations in immune, extracellular matrix, and membrane‐associated pathways across ovarian reserve phenotypes, suggesting differential shifts in the follicular microenvironment linked to altered homeostasis [22, 26]. This multifaceted role of AMH in reflecting and, potentially, modulating the molecular state of the follicular niche is strongly supported by recent evidence. Notably, Vale‐Fernandes et al. demonstrated a tight relationship between circulating and follicular AMH levels and oxidative stress, particularly in patients with altered ovarian dynamics such as polycystic ovary syndrome (PCOS) [27]. Their findings suggest that AMH can serve as a reliable clinical surrogate biomarker of follicular fluid oxidative stress and microenvironment health. This concept aligns with our proteomic and biochemical observations across different AMH thresholds, reinforcing the hypothesis that variations in AMH profiles do not merely quantify the ovarian reserve, but actively mirror specific homeostatic and oxidative imbalances within the oocyte's local environment.

Our results show a specific pattern of positive correlations among immunoglobulin kappa constant (IGKC), immunoglobulin heavy variable 3‐43D (IGHV3‐43D), and adiponectin in follicular fluid in the LAMH group, who exhibit a reduced immune protein profile and a concomitant reduction in adiponectin. This coordinated relationship suggests a potential crosstalk between local metabolism and immune surveillance during folliculogenesis. Adiponectin has potent anti‐inflammatory properties because it reduces the production of pro‐inflammatory cytokines, enhances insulin sensitivity, and promotes a favorable biochemical microenvironment within the ovary, potentially protecting developing oocytes from inflammatory injuries. At the same time, the presence of specific immunoglobulin components, such as IGKC and IGHV3‐43D, suggests physiological, not pathological, immune surveillance essential for tissue remodeling and follicle selection.

Therefore, the biological significance of this complex positive interplay could lie in a shared regulatory mechanism, suggesting a potential link where a decrease in metabolic efficiency (driven by adiponectin) coincides with a decline in the protective immune protein profile. In the LAMH cohort, the concomitant reduction of these three parameters suggests an altered follicular microenvironment. Without the anti‐inflammatory shield of adiponectin, the local tissue is more vulnerable to low‐grade chronic inflammation, while the reduction of structural antibody components like IGKC, which has been associated with oocyte maturation failure—directly reflects an impaired capacity of the granulosa cells to support proper follicular development. Furthermore, these data suggest the existence of a protein “cluster” that may represent a metabolic‐immune pattern associated with a suboptimal follicular microenvironment [28]. IGKC and IGHV3‐43D are the structural components of antibodies, and in FF, the positive correlation confirms that the local immune system is activated in inflammation. Low IGKC levels in FF have been associated in several studies with follicles containing oocytes that fail to mature.

In the LAMH group, a negative correlation was also observed between high lactate levels and low levels of anti‐Müllerian hormone (AMH) in the follicular fluid. This negative correlation may represent a metabolic and hormonal signature associated with diminished ovarian reserve (DOR), potentially reflecting an altered biochemical environment within the follicle. Elevated lactate levels in the follicular fluid have been previously reported in patients with reduced ovarian reserve and endometriosis. This may reflect altered energy metabolism in the follicular microenvironment, which could potentially influence oocyte maturation. The concomitant reduced level of inhibitor of metalloproteinase‐1 (TIMP‐1) may suggest alterations in follicularremodeling, similar to patterns previously described in atretic follicles or follicles with reduced function or impaired follicular maturation [29]. This hypothesis is consistent with the increased level of actin observed in LAMH subjects.

## 5 Conclusion

The main findings of this exploratory pilot study are summarized in Table 3. The results suggest that AMH levels are associated with distinct shifts in the configuration of the follicular microenvironment. In the LAMH group, the downregulation of immunoglobulins and their correlation with adiponectin may indicate a niche characterized by biochemical alterations linked to inflammation. Furthermore, the observed imbalance between lactate and TIMP1 levels may reflect a particular metabolic and proteomic pattern within the follicle. These parameters may represent a defined “cluster” associated with a suboptimal follicular environment, providing a preliminary basis for future studies to investigate these observed exploratory associations in larger clinical cohorts.

**TABLE 3:** Summary of the principal findings.

Category	Finding	Fold change	*p*
Oxidative stress (d‐ROM)	Increased in HAMH	1.66 (HAMH vs LAMH)	< 0.0001
		1.27 (HAMH vs NAMH)	< 0.05
Antioxidant capacity (BAP)	No significant differences among groups	—	—
Lactate	Higher levels in LAMH	1.33 (LAMH vs NAMH)	0.0002
		1.43 (LAMH vs HAMH)	< 0.0001
Adiponectin	Reduced levels in LAMH	0.79 (LAMH vs NAMH)	0.0162
Immunoglobulins	Downregulated in LAMH	# Table 2	< 0.05
HRG, ITIH3, GPLD1	Upregulated in LAMH	# Table 2	< 0.05

Abbreviations: BAP, biological antioxidant potential; GPLD1, phosphatidylinositol‐glycan‐specific phospholipase D; HAMH, high anti‐Müllerian hormone; HRG, histidine‐rich glycoprotein; ITIH3, inter‐alpha‐trypsin inhibitor heavy chain H3; LAMH, low anti‐Müllerian hormone; NAMH, normal anti‐Müllerian hormone.

## 6 Study Limitations

While providing novel insights into the molecular signature of the follicular microenvironment across different ovarian reserve categories, we must acknowledge some limitations in our study. First, the total sample size (*n* = 49) is relatively small. As this is a discovery‐phase pilot study, the findings require validation in larger, independent cohorts to ensure generalizability. Second, performing a formal a priori power analysis was precluded by the exploratory, high‐throughput nature of the proteomic approach.

A major limitation of this study is the absence of direct embryological and clinical outcome data, such as oocyte maturity, fertilization, blastocyst development, and implantation, pregnancy, or live birth rates. Therefore, our observations regarding the follicular microenvironment remain exploratory associations and cannot yet be directly linked to clinical IVF success. Additionally, data on potential confounding factors—including patient BMI, specific stimulation protocols, gonadotropin doses, and the total number of oocytes retrieved—were not fully available for analysis. Furthermore, the clinical criteria for defining PCOS and specific endocrine‐ovulatory disorders, while following the standard clinical practice of the Siena University Hospital, were not strictly standardized within this discovery cohort. Since these variables may independently influence the composition of the follicular fluid, their absence represents a limitation that should be addressed in future research. Furthermore, due to the observational and cross‐sectional design of this study, we must emphasize that the identified relationships between AMH levels, biochemical parameters, and proteomic signatures represent statistical associations rather than definitive causal relationships. Mechanistically, it remains to be elucidated whether altered AMH signaling directly drives the dysregulation of proteins like HRG, ITIH3, and GPLD1, or if these molecular shifts are concurrent, independent reflections of an upstream driving factor, such as accelerated ovarian aging, chronic follicular inflammation, or granulosa cell dysfunction. Consequently, while these biochemical parameters may offer valuable preliminary insights for characterizing distinct ovarian reserve phenotypes, caution should be exercised when inferring direct causality, and mechanistic in vitro or functional models are required to delineate the exact causal pathways.

## 7 Future Perspectives: Clinical Implications, Therapeutic Targets, and Diagnostic Perspectives

To build upon these exploratory findings, specific future validation strategies are highly warranted. First, longitudinal studies should be implemented to track the clinical trajectory of individual oocytes derived from characterized follicular microenvironments. Tracking these oocytes through embryo development via time‐lapse morphokinetics and correlating their molecular profiles with cumulative pregnancy and live birth rates could provide valuable evidence of their prognostic value. Future research could investigate whether the management of female infertility should transcend conventional hormonal stimulation protocols by also focusing on the modulation of the follicular microenvironment. It remains to be investigated whether targeting the reduction of oxidative stress and local inflammation, while monitoring metabolic indicators such as adiponectin and lactate, could successfully support follicular homeostasis and potentially improve reproductive outcomes in assisted reproduction.

Second, interventional approaches are required to determine whether these molecular imbalances can be therapeutically modulated. Future clinical trials could investigate whether targeted pre‐treatment protocols—such as personalized antioxidant supplements to mitigate d‐ROMs or tailored gonadotropin regimes to modulate the local immune‐metabolic niche—might help restore physiological levels of adiponectin, lactate, and structural proteins like HRG and ITIH3, thereby potentially supporting oocyte development and subsequent reproductive outcomes.

The molecular and proteomic alterations unveiled across distinct AMH thresholds may carry potential implications for personalized reproductive medicine. In clinical practice, future research could investigate whether the identified biochemical signatures—such as the immune‐metabolic cluster (adiponectin/immunoglobulins) and the lactate/d‐ROM axis—could be integrated into advanced predictive models. By further exploring these microenvironmental factors in the follicular fluid during oocyte retrieval, future studies could evaluate if these patterns might complement traditional morphological assessment, potentially providing a more comprehensive evaluation of the follicular niche and informing future embryo selection and transfer strategies. Furthermore, these proteomic discoveries may highlight potential pathways for future research aimed at supporting the homeostatic balance of the follicular niche. For instance, it could be hypothesized that the observed upregulation of HRG and the subsequent anti‐angiogenic and pro‐inflammatory pathways might be evaluated in future studies testing the impact of targeted localized anti‐inflammatory agents or specific antioxidant regimens designed to minimize oxidative stress. Similarly, therapies modulating extracellular matrix (ECM) remodeling could be explored to target the hyper‐crosslinking associated with ITIH3 overexpression, potentially reducing follicular stiffness to support tissue fluidity and oocyte release. Lastly, correcting the downregulation of GPLD1 in the High‐AMH phenotype could be investigated as a way to restore crucial paracrine cell‐signaling pathways (e.g., TGF‐β/BMP networks) involved in granulosa cell survival and oocyte maturation. These exploratory findings and hypothetical strategies must be contextualized within the evolving role of AMH in reproductive medicine. As critically evaluated in the recent comprehensive review by Vale‐Fernandes et al. [30], AMH has emerged as a prominent biomarker, even proposed as a core diagnostic criterion for polycystic ovary syndrome (PCOS). Its clinical prominence is driven by unique advantages, including its stability across the menstrual cycle and its unmatched capacity to mirror the true antral follicle count. These attributes strongly validate our choice to stratify patients based on AMH thresholds to capture distinct microenvironmental phenotypes. However, as Vale‐Fernandes et al. also caution, widespread clinical adoption of AMH as a definitive diagnostic tool is still hindered by prominent methodological challenges, most notably substantial assay variability among commercial platforms and the current lack of universally accepted, standardized clinical cut‐offs [30].

Our exploratory findings offer preliminary insights toward addressing this clinical bottleneck; by suggesting that specific AMH cut‐offs (Low vs. Normal vs. High) are associated with distinct functional proteomic and metabolic environments, our study highlights how downstream follicular fluid analysis could potentially complement systemic AMH testing. Overcoming these assay discrepancies through international standardization will be paramount before such candidate molecular signatures could be further evaluated for potential integration into IVF workflows to support personalized clinical decision‐making.

## Funding

This work was supported by the MINISTERO AFFARI ESTERI E DELLA COOPERAZIONE INTERNAZIONALE DELLA REPUBBLICA ITALIANA for the International Cooperation (OcySort_2023 CUP B53C23007370001); Fondazione Cassa di Risparmio di Firenze (COCREIAMO—CUP B13C23003400007).

## Ethics Statement

The authors confirm that all ethical considerations are thoroughly addressed. The study was compliant with the Declaration of Helsinki and approved by the ethics committee of the University of Siena (CEAVSE ethics statement, Prot. No. 11161_2017). All procedures followed were in accordance with the ethical standards of the responsible committee on human experimentation (institutional and national) and with the Helsinki Declaration of 1964 and its later amendments.

## Conflicts of Interest

The authors declare no conflicts of interest.

## Supporting information


**Table S1:** (A) LC‐ESI_MS/MS analysis data. (B) Classification of the differentially expressed proteins into Low, Normal, and High anti‐Müllerian hormone (AMH) groups.

## Data Availability

The data that support the findings of this study are available on request from the corresponding author. The data are not publicly available due to privacy or ethical restrictions.
